# Long-term maternal health outcomes following autologous in vitro fertilization compared with medically unassisted conceptions and with nulliparas

**DOI:** 10.1007/s10815-026-03957-4

**Published:** 2026-07-06

**Authors:** Hila Shalev-Ram, Amir Wiser, Roni Rahav-Koren, Einat Haikin-Herzberger, Roei Shlezinger, Mattan Levi, Netanella Miller

**Affiliations:** 1 IVF Unit, Department of Obstetrics and Gynecology, Meir Medical Center, Kfar Saba, Israel; 2 School of Medicine, Faculty of Health and Medical Sciences, Tel Aviv University, Tel Aviv, Israel; 3 Department of Statistics and Data Science, Hebrew University of Jerusalem, Jerusalem, Israel; 4 Department of Obstetrics and Gynecology, Mayanei Hayeshua Medical Center, Bnei Brak, Israel

**Keywords:** In vitro fertilization, Nulliparous, Medically unassisted conceptions, Long-term morbidity, Thyroid diseases, Parity

## Abstract

**Purpose:**

The use of in vitro fertilization (IVF) has increased substantially, raising interest in the long-term health outcomes of women undergoing these treatments.

**Objective:**

To assess the association between conception through autologous IVF and the long-term incidence of chronic diseases, compared with two reference groups: women who conceived through medically unassisted conception (UC) and age-matched nulliparous women.

**Methods:**

This retrospective cohort study used electronic health records from a large health maintenance organization and included women who delivered before age 45 between 2000 and 2018. Three pairwise comparisons were performed: IVF mothers versus UC mothers, IVF mothers versus nulliparous women, and UC mothers versus nulliparous women. For the IVF versus UC comparison, each IVF mother was matched to a UC mother by age and parity at delivery. For comparisons with nulliparous women, controls were matched by age according to the mother’s age at first delivery. The delivery date served as the index date for follow-up. Primary outcomes were chronic diseases diagnosed after the index pregnancy and persisting for at least three years.

**Results:**

The study included 17,425 IVF mothers matched to 17,425 UC mothers, 10,358 IVF mothers matched to 10,358 nulliparous women, and 38,810 UC mothers matched to 38,810 nulliparous women. Compared with UC, IVF was associated with a higher risk of thyroid disease (HR 1.11, *p* < 0.05). Compared with nulliparous women, IVF mothers had higher risks of thyroid disease (HR 2.07), rheumatologic disease (HR 1.87), neurologic disease (HR 2.51), and psychiatric conditions (HR 1.34) (all *p* < 0.05). UC mothers also had higher risks of thyroid disease (HR 1.67), rheumatologic disease (HR 1.79), cardiology disease (HR 1.47), and psychiatric conditions (HR 1.47) compared with nulliparous women (all *p* < 0.05).

**Conclusions:**

Long-term health outcomes after autologous IVF were largely comparable to those after UC, except for a modestly increased risk of thyroid disease. Compared with nulliparous women, both IVF and UC mothers had higher risks of several chronic conditions, suggesting that pregnancy itself may contribute to long-term maternal health trajectories. Thyroid function monitoring may be considered for women who conceive through autologous IVF.

## Introduction

Since the first successful in vitro fertilization (IVF) birth in 1978, the use of assisted reproductive technology has increased substantially, driven in part by improving success rates and delayed childbearing at more advanced maternal ages [1, 2]. As IVF use expands, its long-term implications for maternal health have become an important public health question. Most prior research has focused on short-term maternal outcomes, including ovarian hyperstimulation syndrome, hypertensive disorders of pregnancy, gestational diabetes, placental complications, preterm birth, cesarean delivery, and severe maternal morbidity such as ICU admission, transfusion, and hysterectomy [3–12] IVF has also been associated with increased risk for postpartum stroke, with higher rates during delivery hospitalization and within 12 months postpartum, possibly related to hypertensive complications after frozen embryo transfer [13–17] In contrast, data on long-term maternal health after IVF remain sparse. A meta-analysis of more than 41,000 women found no differences in long-term cardiovascular event rates between ART and unassisted conception groups [9]. However, other chronic maternal outcomes remain poorly defined. These findings raise the possibility that IVF treatment, the underlying infertility diagnosis, or both may reflect broader long-term health vulnerability. However, the available evidence is limited and inconsistent.

A major challenge in interpreting prior studies is separating risks related to IVF treatment from those related to pregnancy itself. Women who undergo IVF differ from the general obstetric population in age, parity, health behaviors, fertility and comorbidity burden, complicating causal inference [18–20]. Underlying infertility diagnoses, such as polycystic ovary syndrome, endometriosis, diminished ovarian reserve, or premature ovarian insufficiency, may themselves be associated with later chronic disease risk [21–24]. In addition, factors that contribute to infertility, including obesity, insulin resistance, metabolic dysfunction, endometriosis and advanced maternal age, may also independently contribute to long-term maternal morbidity [21, 24, 25]. Most studies compare IVF pregnancies only with medically unassisted conceptions (UC) and few include nulliparous women as a reference group. This limits the ability to distinguish risks associated with ART from those associated with pregnancy.

To address these gaps, we conducted a large, population-based, cohort study of long-term maternal health outcomes after autologous IVF. We compared women who conceived via IVF with two matched reference groups: women with UC and nulliparous women. We also compared women with UC to nulliparous women to better assess the contribution of pregnancy itself. This design allowed a more precise evaluation of the respective roles of IVF treatment, underlying infertility, and pregnancy in long-term maternal health outcomes.

## Methods

### Study design

This retrospective cohort study compared the incidence of chronic diseases among mothers who conceived via IVF with autologous oocytes, mothers with UC, and nulliparous women. Data were obtained from the electronic medical records of Maccabi Healthcare Services, a large integrated care organization in Israel, serving 2.3 million individuals (approximately 25% of the pregnant population). The study includes up to 18 years of follow-up after the index delivery, allowing evaluation of long-term maternal health trajectories.

### Study population

Eligible participants were women who delivered between 2000 and 2018 and were younger than 45 years of age. Each woman contributed only once to the analysis. For women with more than one eligible pregnancy during the study period, the first eligible delivery was selected as the index pregnancy, and subsequent pregnancies were not analyzed as separate observations. This approach ensured that individuals were not duplicated in the cohort.

The IVF group included pregnancies conceived using autologous oocytes following conventional IVF or intracytoplasmic sperm injection (ICSI), including both fresh embryo transfer and frozen embryo transfer (FET) cycles. Pregnancies conceived using donor oocytes and pregnancies following preimplantation genetic testing (PGT) were not included.

The study population included mothers after autologous IVF, mothers after UC, and nulliparous women as controls. Women without adequate follow-up data were excluded. Women were also excluded if they had any documented diagnosis, before fertility treatment or before the index pregnancy, of any of the chronic diseases assessed as study outcomes. This exclusion was limited to the chronic disease categories evaluated in the study and did not refer to unrelated medical conditions. Nulliparous women with infertility diagnoses were also excluded.

For IVF–UC comparisons, each IVF mother was matched to a woman of the same age and parity at delivery. For comparisons with nulliparous women, including both IVF versus nulliparous women and UC versus nulliparous women, matching was based solely on age, using the IVF or UC mother’s age at her first delivery as the reference. The delivery date of the relevant IVF or UC pregnancy served as the index date for follow-up. For the matched nulliparous women, who had no delivery date, this same matched delivery date was assigned as the index date. Nulliparous women were included only if they were registered members of the health maintenance organization and had available longitudinal medical records from the assigned index date onward.

### Primary outcomes

The primary outcomes were chronic diseases diagnosed after the index pregnancy and persisting for at least three years. Diagnoses were identified using International Classification of Diseases, 9th Revision, Clinical Modification (ICD-9-CM) codes recorded in Maccabi Healthcare Services electronic medical records, including both hospital-based records and community-based outpatient clinic records. Conditions assessed included cardiac diseases such as congestive heart failure (428), atrial fibrillation (427), ischemic heart disease (410–414), and valve disease (396, 397, 424); cardiovascular diseases including cerebrovascular accident (429.2), transient ischemic attack (435.9), and peripheral vascular disease (443.9); diabetes mellitus (249–250); hypertension (401–405); inflammatory bowel disease (555–556); rheumatologic diseases including systemic lupus erythematosus (710), systemic sclerosis (710), sicca syndrome (710), dermatomyositis (710), polymyositis (710), pyogenic arthritis (711), Behçet’s syndrome (714), rheumatoid arthritis (714), osteoarthritis (715), and polymyalgia rheumatica (725); immunologic diseases (279); thyroid diseases (240–246); and psychiatric disorders (290–319). Cancer diagnoses were excluded because they were addressed in a separate study.

### Covariates and data collection

Maternal demographic and clinical characteristics included socioeconomic status (SES), marital status, age at delivery, number of children, BMI, smoking status, and use of hormone replacement therapy. SES was determined using the national census poverty index of the mother’s residential enumeration area, incorporating education level, employment, and standard of living, with scores ranging from 1 (lowest) to 20 (highest). Obesity was defined as BMI greater than 30 kg/m^2^.

### Ethics

The study was approved by the Institutional Review Board (IRB #0046–18-BBL), and informed consent was waived due to the retrospective design and the use of deidentified data. All data were fully anonymized before analysis.

### Statistical analysis

Discrete variables were presented as counts and percentages, and continuous variables as means with standard deviations. Group comparisons were performed using t-tests or chi-square tests, as appropriate. Matching criteria included age at delivery and parity for IVF versus UC comparisons, and age alone for IVF versus nulliparous comparisons and UC versus nulliparous comparisons.

Risk-time analyses extended from the index date to the first diagnosis of a chronic disease, death, or end of follow-up. Cox proportional hazards models were used to estimate adjusted hazard ratios for disease and mortality risk, adjusting for age, number of children, and smoking status. A two-sided p-value of less than 0.05 was considered statistically significant. Analyses were performed using SPSS® version 24.0 and Python 3.7.9.

## Results

The study included 17,425 women who conceived via autologous IVF, who were matched to 17,425 women with UC, 10,358 women who conceived via IVF matched to 10,358 nulliparous women, and 38,810 women with UC matched to 38,810 nulliparous women. Mean follow-up time was 10.0 ± 4.1 years among women who conceived via autologous IVF and 6.4 ± 4.1 years among women with medically unassisted conception. Mean follow-up time was not available for the nulliparous comparison group.

As shown in Table 1, age at first delivery was identical across matched groups. Obesity was more common in the IVF group than in the UC group (13.6% vs. 9.4%, *p* = 0.001), but less common than in nulliparous women (13.4% vs. 16.6%, *p* = 0.001). Women who conceived via IVF had slightly higher SES than those with UC (11.0 vs. 10.4, *p* = 0.001), but slightly lower SES than nulliparous women (10.7 vs. 11.2, *p* = 0.001). Smoking was more common in the IVF group than in the UC group (4.3% vs. 2.8%, *p* = 0.001), but less common than among nulliparous women (4.3% vs. 21.0%, *p* = 0.001). HRT use was rare and similar in the IVF and UC groups (0.04% vs. 0.01%, *p* = 0.114), but more frequent among nulliparous women (0.7% vs. 0.1%, *p* = 0.001). Single marital status was more common in the IVF group than in the UC group (41.8% vs. 38.0%, *p* = 0.001), and more common among nulliparous women (88.7% vs. 41.2%, p < 0.001).

**Table 1:** Characteristics of mothers after conception with IVF compared to mothers after medically unassisted conception (UC) and nulliparous women

Characteristic*	IVF (*N* = 17425)	UC (*N* = 17425)	*P*-Value	IVF (*N* = 10358)	Nulliparous (*N* = 10358)	*P*-Value	UC (*N* = 38810)	Nulliparous (*N* = 38810)	*P*-Value
SES	11 ± 5.3	10.4 ± 5.6	0.001	10.7 ± 5.3)	11.2 ± (4.8)	0.001	7.9 ± (4.6)	10.5 ± (5.1)	0.001
Age at first delivery (years)	33.4 ± 5.1	33.4 ± 5.1	1	32.1 ± 5.6	32.1 ± 5.6	1	24.6 ± 6	24.6 ± 6	1
Smoking	753 (4.3)	489 (2.8)	0.001	450 (4.3)	2190 (21)	0.001	1682 (4.3)	7223 (18.6)	0.001
Use of HRT	8 (0.04)	2 (0.01)	0.114	7(0.1)	69 (0.7)	0.001	37 (0.1)	180 (0.5)	0.001
Obesity (BMI > 30)	2363 (13.6)	1644 (9.4)	0.001	1384 (13.4)	1721 (16.6)	0.001	3397 (8.8)	4558 (11.7)	0.001
Family status, single	7279 (41.8)	6777 (38)	0.001	4246 (41.2)	9184 (88.7)	< 0.001	16,543 (42)	36,044 (92)	0.001

Data are presented as mean ± SD or n (%). *IVF* in vitro fertilization, *UC* medically unassisted conception, *SES* socioeconomic status, *SD* standard deviation, *HRT* hormone replacement therapy, *BMI* body mass index. *P*-values were calculated using t-tests for continuous variables and chi-square tests for categorical variables, as appropriate

Compared with women with UC, nulliparous women had higher SES (10.5 vs. 7.9, *p* = 0.001), and higher rates of smoking (18.6% vs. 4.3%, *p* = 0.001), HRT use (5.0% vs. 0.1%, *p* = 0.001), obesity (11.7% vs. 8.8%, *p* = 0.001), and single status (92.0% vs. 42.0%, *p* = 0.001).

Long-term health outcomes are summarized in Table 2. Compared with the IVF group, the UC group had higher rates of rheumatologic (6.0% vs. 4.9%, *p* = 0.001) and psychiatric disorders (10.0% vs. 9.1%, *p* = 0.004), whereas endocrinologic disorders were more common in the IVF group (2.2% vs. 1.3%, *p* = 0.001).

**Table 2:** Long-term outcomes of mothers after conception via IVF compared to mothers after medically unassisted conception (UC) and nulliparous women

Long Term outcomes *N* (%)	IVF (*N* = 17425)	UC (*N* = 17425)	*P*-Value	IVF (*N* = 10358)	Nulliparous (*N* = 10358)	*P*-Value	UC (*N* = 38810)	Nulliparous (*N* = 38810)	*P*-Value
Cardiology (all)	331 (1.9)	334 (1.9)	0.938	201 (1.9)	172 (1.7)	0.143	495 (1.3)	548 (1.4)	0.105
Cardiovascular disease	29 (0.2)	33 (0.2)	0.703	14 (0.1)	25 (0.2)	0.109	37 (0.1)	34 (0.1)	0.812
Diabetes mellitus	403 (2.3)	368 (2.1)	0.216	211 (2)	224 (2.2)	0.562	760 (2)	81 (0.2)	0.001
Hypertension	940 (5.4)	945 (5.4)	0.925	504 (4.9)	353 (3.4)	0.001	883 (2.3)	599 (1.5)	0.001
Inflammatory bowel disease	164 (0.9)	158 (0.9)	0.789	88 (0.8)	123 (1.2)	0.004	259 (0.7)	361 (0.9)	0.001
Rheumatology	854 (4.9)	1048 (6)	0.001	470 (4.5)	147 (1.4)	0.001	1034 (2.7)	349 (0.9)	0.001
Neurology	76 (0.4)	75 (0.4)	1	43 (0.4)	19 (0.2)	0.004	97 (0.2)	47 (0.1)	0.001
Psychiatric Conditions	1590 (9.1)	1749 (10)	0.004	901 (8.7)	384 (4.2)	0.001	2874 (7.4)	1263 (3.5)	0.001
Immunology	51 (0.3)	48 (0.3)	0.840	27 (0.3)	6 (0.06)	0.001	66 (0.2)	24 (0.1)	0.001
Thyroid diseases	3078 (17.7)	3000 (17.2)	0.277	1808 (17.5)	448 (4.8)	0.001	4659 (12)	1334 (3.7)	0.001
Nephrology	12 (0.1)	11 (0.1)	1	7 (0.07)	1 (0.01)	0.07	17 (0.4)	3 (0.07)	0.004
Pulmonary	6 (0.03)	9 (0.05)	0.605	1 (0.01)	5 (0.04)	0.219	10 (0.02)	12 (0.03)	0.831
Hematology	11 (0.1)	13 (0.1)	0.838	6 (0.06)	2 (0.02)	0.289	24 (0.1)	5 (0.012)	0.001
Death	44 (0.3)	48 (0.3)	0.754	28 (0.3)	22 (0.2)	0.479	51 (0.1)	33 (0.1)	0.063

Values are presented as n (%). *UC* medically unassisted conception, *IVF* in vitro fertilization. “Cardiology (all)” includes congestive heart failure, atrial fibrillation, ischemic heart disease, valve disease, cerebrovascular accident, transient ischemic attack, and peripheral vascular disease. “Psychiatric conditions” includes substance-related disorders, depression, anxiety, intellectual disability and developmental delay, attention-deficit/hyperactivity disorder, eating disorders, and schizophrenia

Compared with nulliparous women, women who conceived via IVF had higher rates of hypertension (4.9% vs. 3.4%, *p* = 0.001), rheumatologic disease (4.5% vs. 1.4%, *p* = 0.001), neurologic disorders (0.4% vs. 0.2%, *p* = 0.004), psychiatric disorders (8.7% vs. 4.2%, *p* = 0.001), immunologic disease (0.3% vs. 0.006%, *p* = 0.001), and thyroid disease (17.5% vs. 4.8%, *p* = 0.001). Inflammatory bowel disease was less common in the IVF group (0.8% vs. 1.2%, *p* = 0.004).

Similarly, compared with nulliparous women, the UC group had higher rates of diabetes mellitus (2.0% vs. 0.2%, *p* = 0.001), hypertension (2.3% vs. 1.5%, *p* = 0.001), rheumatologic disease (2.7% vs. 0.9%, *p* = 0.001), neurologic disorders (0.2% vs. 0.1%, *p* = 0.001), psychiatric disorders (7.4% vs. 3.5%, *p* = 0.001), immunologic disease (0.2% vs. 0.1%, *p* = 0.001), nephrological disease (0.4% vs. 0.07%, *p* = 0.004), and thyroid disease (12.0% vs. 3.7%, *p* = 0.001). Inflammatory bowel disease was less common in the UC group than in nulliparous women (0.7% vs. 0.9%, *p* = 0.001).

**Fig. 1:**
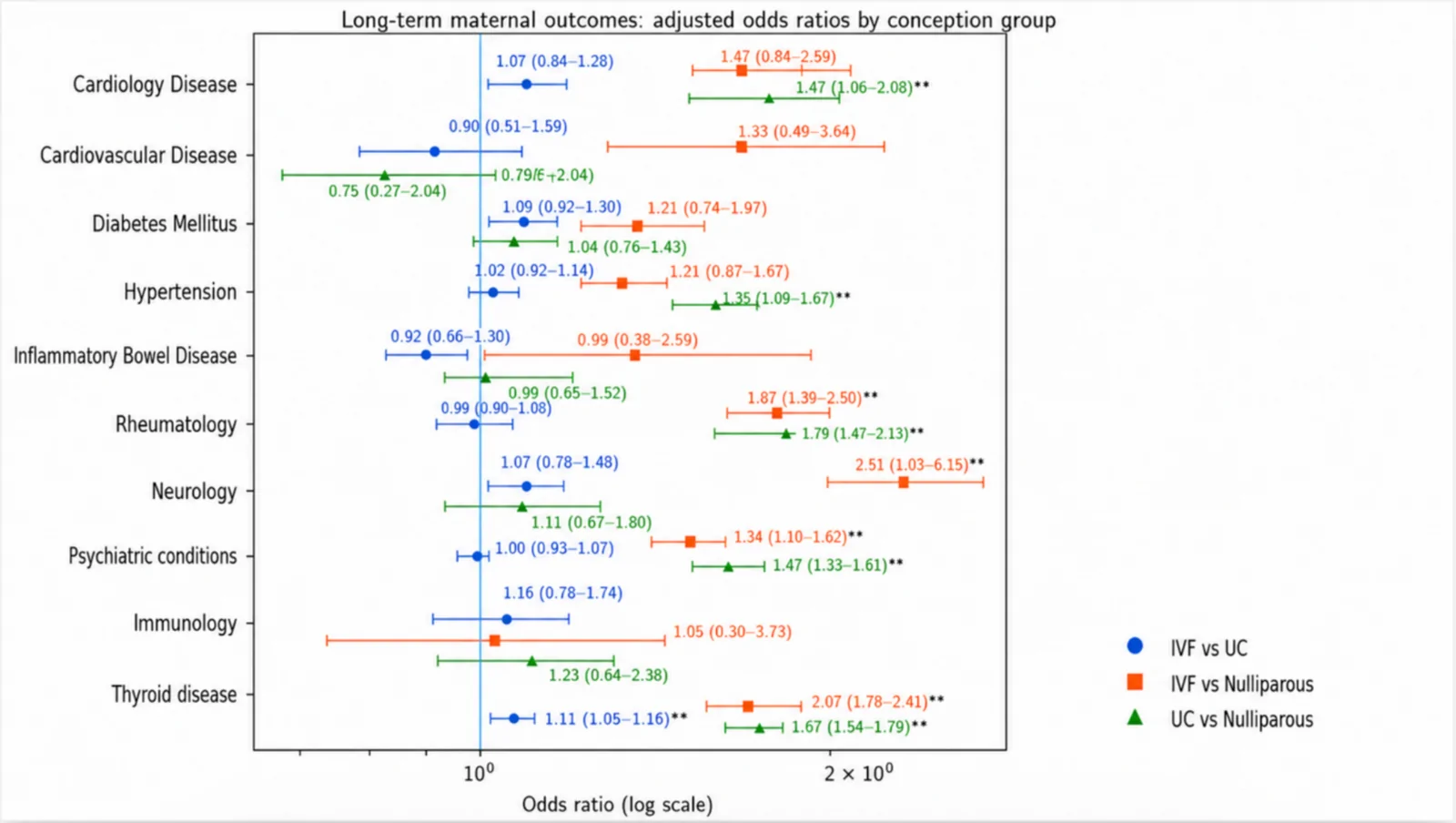
Dot plot for hazard ratios for comparison between IVF and UC, IVF and nulliparous and UC and Nulliparous. *Adjusted for age, number of children, and smoking/UC = medically unassisted conception

Multivariable Cox regression adjusted for maternal age, smoking, and parity (Table 3) showed that, compared with women with UC, IVF mothers had a higher risk of thyroid disease (HR 1.11, 95% CI 1.05–1.16). Compared with nulliparous women, IVF mothers had higher risks of thyroid disease (HR 2.07, 95% CI 1.78–2.41), rheumatologic disease (HR 1.87, 95% CI 1.39–2.50), neurologic disorders (HR 2.51, 95% CI 1.03–6.15), and psychiatric conditions (HR 1.34, 95% CI 1.10–1.62). Women with UC also had higher risks of thyroid disease (HR 1.67, 95% CI 1.54–1.79), rheumatologic disease (HR 1.79, 95% CI 1.47–2.13), cardiology disease (HR 1.47, 95% CI 1.06–2.08), hypertension (HR 1.35, 95% CI 1.09–1.67), and psychiatric conditions (HR 1.47, 95% CI 1.33–1.61) compared with nulliparous women (Fig. 1).

**Table 3:** Cox regression of long-term maternal outcomes: pairwise comparisons between IVF, unassisted conception (UC), and nulliparous women

Long-term outcomes	IVF vs. UC	IVF vs. Nulliparous	UC vs. Nulliparous
Cardiology (all)	1.07 (0.84–1.28)	1.47 (0.84–2.59)	1.47 (1.06–2.08)**
Cardiovascular disease	0.90 (0.51–1.59)	1.33 (0.49–3.64)	0.75 (0.27–2.04)
Diabetes mellitus	1.09 (0.92–1.30)	1.21 (0.74–1.97)	1.04 (0.76–1.43)
Hypertension	1.02 (0.92–1.14)	1.21 (0.87–1.67)	1.35 (1.09–1.67)**
Inflammatory bowel disease	0.92 (0.66–1.30)	0.99 (0.38–2.59)	0.99 (0.65–1.52)
Rheumatology	0.99 (0.90–1.08)	1.87 (1.39–2.50)**	1.79 (1.47–2.13)**
Neurology	1.07 (0.78–1.48)	2.51 (1.03–6.15)**	1.11 (0.67–1.85)
Psychiatric conditions	1.00 (0.93–1.07)	1.34 (1.10–1.62)**	1.47 (1.33–1.61)**
Immunology	1.16 (0.78–1.74)	1.05 (0.30–3.73)	1.23 (0.64–2.38)
Thyroid disease	1.11 (1.05–1.16)**	2.07 (1.78–2.41)**	1.67 (1.54–1.79)**
Death	NA	NA	NA

Values represent adjusted hazard ratios with 95% confidence intervals from Cox proportional hazards models. Models were adjusted for age at first delivery, number of children, and smoking status. IVF, in vitro fertilization; UC, medically unassisted conception; NA, not applicable. “Cardiology (all)” includes congestive heart failure, atrial fibrillation, ischemic heart disease, valve disease, cerebrovascular accident, transient ischemic attack, and peripheral vascular disease. **p < 0.05

## Discussion

### Main findings

This large, retrospective, cohort study compared the long-term health outcomes of women who conceived without medical assistance, via IVF and nulliparous women. The findings indicated that women after IVF had a higher long-term risk of thyroid disease than both women with UC and nulliparas. Compared with nulliparous women, IVF mothers also had higher risks of rheumatologic, neurologic, and psychiatric disorders, independent of age at first delivery, smoking, and parity. Women with UC similarly had higher risks of thyroid, rheumatologic, cardiologic, and psychiatric disease than did nulliparous women.

The association between IVF and subsequent thyroid disease is biologically plausible. Women with diminished ovarian reserves or premature ovarian insufficiency, both common indications for IVF, have higher rates of thyroid autoimmunity [26, 27]. Geva et al. likewise reported a higher prevalence of antithyroid antibodies in infertile women than in healthy nulligravid controls, suggesting an underlying predisposition [28]. IVF treatment itself may also contribute. Controlled ovarian stimulation can transiently increase TSH and alter thyroid-binding globulin and free thyroid hormone levels [29] and long-acting GnRH agonists have been associated with new-onset or previously unrecognized autoimmune thyroid disease [30–32], possibly through GnRH receptors expressed in thyroid tissue [33]. Together, these mechanisms may help explain the persistently elevated thyroid risk observed after IVF.

The comparator-group pattern is also informative. Compared with nulliparous women, thyroid disease risk was approximately twice as high after IVF and 67% higher after unassisted conception, whereas IVF was associated with only an 11% higher risk than UC. This gradient suggests that pregnancy itself may contribute to long-term thyroid risk, with IVF and/or the underlying infertility adding excess risk. This interpretation is consistent with thyroid physiology associated with pregnancy. Pregnancy is accompanied by major hormonal and immunologic changes, and the postpartum period is marked by immune rebound, which may unmask latent thyroid autoimmunity and trigger postpartum thyroiditis [34–36]. Postpartum thyroiditis is reported in approximately 5%–10% of postpartum women, with higher rates in selected high-risk populations, and typically occurs 1–4 months after delivery [37]. Although it is frequently transient, approximately 25% of women progress to permanent hypothyroidism within 3–5 years, and recurrence in subsequent pregnancies is not uncommon [35, 37, 38] (37)Overall, these findings suggest that pregnancy may increase long-term thyroid risk, while the stronger association observed after IVF points to an additional contribution from IVF treatment, underlying infertility, or both.

The role of parity in long-term maternal morbidity may partly explain the higher risks observed among both IVF and UC mothers compared with nulliparous women. Prior studies suggest that the association between pregnancy and later autoimmune disease is heterogeneous and varies by disease subtype and postpartum timing. In a large Danish cohort, childbirth was associated with only a small increase in overall autoimmune disease risk, but with a modestly higher risk of female-predominant autoimmune diseases, including Graves’ disease, Hashimoto thyroiditis, sarcoidosis, psoriasis, and erythema nodosum [39]. The postpartum period may be particularly relevant, as immune rebound and a more pro-inflammatory milieu may trigger or unmask latent autoimmune disease, especially during the first year after delivery [40–43] Pregnancy complications have also been linked to later autoimmune disease, although whether this reflects causality or previously unrecognized disease remains uncertain [42]. Overall, these data support the possibility that pregnancy and parity, rather than IVF or infertility alone, contribute to selected long-term outcomes, particularly thyroid and rheumatologic morbidity.

Pregnancy may contribute to long-term rheumatologic risk. In our cohort, both IVF and UC were associated with a higher risk of rheumatologic disease than nulliparity, whereas no excess risk was observed for IVF versus UC, supporting a pregnancy-related effect. The postpartum period is characterized by immune and inflammatory changes [44], and new-onset rheumatoid arthritis has been reported to occur disproportionately after pregnancy, with an approximately fivefold increased postpartum risk [45–47]. The somewhat stronger association observed after IVF may also suggest an additional contribution of infertility and/or its treatment. Women with infertility more often harbor autoimmune antibodies even without overt disease [48], and infertility itself has been linked to later systemic autoimmune rheumatic disease [49–51]. ART-related immune modulation may further contribute, as autoantibodies have been associated with adverse IVF outcomes, particularly miscarriage, although the evidence remains limited and heterogeneous [52].. An older study also reported increasing zona pellucida antibody prevalence after repeated IVF failures, suggesting possible autoimmune activation after repeated ovarian intervention, but these findings should be interpreted cautiously [53].

Psychiatric morbidity showed a distinct pattern. No excess risk was observed in the IVF versus UC comparison (HR 1.00), whereas both IVF and UC were associated with higher long-term psychiatric risk than was nulliparity (HR 1.34 and 1.47, respectively). This pattern suggests that pregnancy itself, rather than IVF treatment alone, may be a major contributor to later psychiatric morbidity. Consistent with this, a longitudinal Finnish study found very similar psychotropic use trajectories among women who achieved live birth after IVF and those who conceived without medical assistance, from 3 years before conception to 12 years afterward, suggesting that once pregnancy and live birth occur, long-term psychiatric outcomes are similar regardless of conception method [54].The higher psychiatric morbidity observed in both parous groups compared with nulliparous women is biologically and clinically plausible. The postpartum period is a well-recognized window of vulnerability, with depression affecting approximately 6%–13% of women in the first 3 months after delivery. This risk is linked to rapid hormonal shifts, obstetric complications, negative birth experiences, sleep deprivation, caregiving burden, and preexisting psychiatric vulnerability, and is especially pronounced in women with prior mood or anxiety disorders or untreated antenatal depression [55–57].

The risk for neurologic morbidity was significantly higher in women who conceived via IVF than in nulliparas (HR 2.51), whereas no significant increase was observed in the IVF versus UC comparison (HR 1.07) or the UC versus nulliparous comparison (HR 1.11). This pattern suggests that the excess neurologic risk may be more closely related to characteristics of women requiring IVF than to pregnancy itself.

This interpretation is biologically plausible. Several neurologic disorders are associated with reduced fertility in women through disruption of hypothalamic-pituitary–gonadal function, menstrual irregularities, anovulation, sexual dysfunction, physical disability, and treatment-related effects. Epilepsy is the best-established example, as both seizure activity and antiepileptic medications can impair reproductive endocrine function and increase the risk of polycystic ovary syndrome and early menopause [1, 2]. Other neurologic conditions, including multiple sclerosis and myasthenia gravis, may also reduce fertility through physical limitations, fatigue, sexual dysfunction, and concerns regarding pregnancy or medication safety [58, 59]. Women with neurologic disability, are likewise more likely to experience infertility and less likely to seek infertility care [60].

Taken together, these findings suggest that the higher long-term neurologic morbidity observed after IVF may reflect underlying neurologic or neuroendocrine vulnerability among women requiring fertility treatment, rather than an effect of pregnancy alone. This interpretation is further supported by evidence that infertility itself does not appear to increase the risk of developing multiple sclerosis, arguing against reverse causation in at least some neurologic conditions [61].

Cardiovascular findings also support a pregnancy-related effect. Cardiology disease risk did not differ meaningfully between IVF and UC (HR 1.07), but was similarly elevated in both groups compared with nulliparous women (both HR 1.47). Hypertension showed the same overall pattern, with little difference between IVF and UC (HR 1.02) and higher risk in both groups relative to nulliparity (HR 1.21 for IVF and 1.35 for UC). These results suggest that pregnancy itself, rather than IVF treatment, may underlie the increased long-term risk of cardiovascular disease and hypertension. This is biologically plausible, given the marked hemodynamic and vascular changes of pregnancy and the established link between hypertensive disorders of pregnancy and later chronic cardiovascular disease [62–64].

## Strengths and limitations

This investigation had several important strengths. It was based on a very large cohort with up to 18 years of follow-up and detailed electronic health records. Importantly, to our knowledge, this is the first study to evaluate long-term chronic disease outcomes in women who conceived via autologous IVF compared with women who conceived without medical assistance and with nulliparous women, while also directly comparing US with nulliparity. Adjustment for key confounders, including age at first delivery, parity, and smoking status, further strengthened the validity of the findings. This design allowed a nuanced assessment of the relative contributions of IVF treatment, underlying subfertility, and pregnancy to long-term maternal health outcomes.

Certain limitations should also be acknowledged, although they should be interpreted with caution. Some clinical variables, such as the specific indication for IVF and the medications used during treatment, were not available. In addition, although BMI and socioeconomic status were available for descriptive analyses, adjustment for these variables in the multivariable models was limited by data completeness. Therefore, residual confounding by BMI, socioeconomic status, and other unmeasured clinical or lifestyle factors cannot be excluded. The retrospective design also did not allow differentiation between fresh and frozen cycles, which may have introduced some variability.

The severity of chronic conditions could not always be assessed, and some diagnoses may have been under-recorded; however, this limitation applied uniformly across all study groups. A degree of surveillance bias was possible, particularly for thyroid disease, since IVF patients may undergo more frequent medical evaluations. Even so, the comprehensive documentation system of our health organization, where every diagnosis is time-stamped and recorded, supports the overall reliability and consistency of the data.

Mean follow-up time was not available for the nulliparous comparison group, which should be considered when interpreting comparisons involving nulliparous women.

Another limitation is that the current analysis did not assess whether chronic disease risk varied over time. The models estimated overall long-term associations and did not account for potential time-varying effects, such as whether risk was higher during the first 5 years after the index date and then attenuated or increased later in follow-up. Future studies using time-stratified analyses or models that allow for non-proportional hazards may better characterize the temporal pattern of risk after IVF, unassisted conception, and nulliparity.

## Conclusions

Autologous IVF was associated with increased long-term thyroid morbidity compared with both medically unassisted conception and nulliparity. Compared with nulliparous women, both IVF and unassisted conception were associated with higher risks of rheumatologic disease, psychiatric morbidity, and thyroid disease, supporting a contribution of pregnancy itself to later maternal morbidity. The additional excess of thyroid disease after IVF, and the higher neurologic risk seen only in the IVF versus nulliparous comparison, suggest that IVF treatment, underlying infertility, or both may further contribute to selected long-term health risks.

## Data Availability

The data that support the findings of this study are not publicly available due to institutional and privacy restrictions but are available from the corresponding author upon reasonable request and subject to approval by the relevant authorities.
